# Whole-genome sequencing and pathogenicity analysis of *Rhodococcus equi* isolated in horses

**DOI:** 10.1186/s12917-024-04167-9

**Published:** 2024-08-12

**Authors:** Bin Hu, Sichao Gao, Hao Zhang, Qiaoqiao Li, Gaojian Li, Shuairan Zhang, Yanan Xing, Yanyi Huang, Shuyi Han, Ying Tian, Wei Zhang, Hongxuan He

**Affiliations:** 1 College of Animal Science and Veterinary Medicine, Henan Institute of Science and Technology, Xinxiang, China; 2 CAS Key Laboratory of Animal Ecology and Conservation Biology, Institute of Zoology, Chinese Academy of Sciences, Beijing, China; 3 University of Chinese Academy of Sciences, Beijing, China; 4 College of Shenyang Institute of Technology, Shenyang, Liaoning China; 5 Anhui University of Science and Technology, Huainan, China; 6 Beijing Wildlife Rescue and Rehabilitation Center, Beijing, China

**Keywords:** *Rhodococcus equi*, Genome, Gene islands, Virulence factors, Drug resistance genes, Secretion systems

## Abstract

**Background:**

*Rhodococcus equi* (*R. equi*) is a Gram-positive zoonotic pathogen that frequently leads to illness and death in young horses (foals). This study presents the complete genome sequence of *R. equi* strain BJ13, which was isolated from a thoroughbred racehorse breeding farm in Beijing, China.

**Results:**

The BJ13 genome has a length of 5.30 Mb and consists of a complete chromosome and a plasmid measuring 5.22 Mb and 0.08 Mb, respectively. We predicted 4,929 coding gene open reading frames, along with 52 tRNAs and 12 rRNAs. Through analysis of mobile genetic elements, we identified 6 gene islands and 1 prophage gene. Pathogenic system analysis predicted the presence of 418 virulence factors and 225 drug resistance genes. Secretion system analysis revealed the prediction of 297 secreted proteins and 1,106 transmembrane proteins. BJ13 exhibits genomic features, virulence-associated genes, potential drug resistance, and a virulence plasmid structure that may contribute to the evolution of its pathogenicity. Lastly, the pathogenicity of the isolated strain was assessed through animal experiments, which resulted in inflammatory reactions or damage in the lungs, liver, and spleen of mice. Moreover, by the 7th day post-infection, the mortality rate of the mice reached 50.0%, indicating complex immune regulatory mechanisms, including overexpression of IL-10 and increased production of pro-inflammatory cytokines like TNF-α. These findings validate the strong pathogenicity of the isolated strain and provide insights for studying the pathogenic mechanisms of *Rhodococcus equi* infection.

**Conclusions:**

The complete genome sequence of *R. equi* strain BJ13 provides valuable insights into its genomic characteristics, virulence potential, drug resistance, and secretion systems. The strong pathogenicity observed in animal experiments underscores the need for further investigation into the pathogenic mechanisms of *R. equi* infection.

**Supplementary Information:**

The online version contains supplementary material available at 10.1186/s12917-024-04167-9.

## Introduction

*Rhodococcus equi* (*R. equi*) is a Gram-positive zoonotic pathogen with a high G + C content that causes pulmonary and extrapulmonary pyogenic granulomatous infections in various animal species, including humans [1, 2]. *R equi* parasitizes host macrophages and evades killing by inhibiting the fusion of phagosomes/autophagosomes and lysosomes, resulting in lung tissue destruction and neutrophil influx, eventually leading to necrotizing pneumonia [3–5]. Foals between 1 and 6 months of age are particularly susceptible to *R. equi* infection, which is globally distributed and a significant cause of morbidity and mortality in foals [6].

The pathogenicity of *R. equi* relies on the presence of a large virulence plasmid, and the pathogenicity islands (PAIs) carried by this plasmid greatly influence the interaction between *R. equi* and its host [7, 8]. *R. equi* infects its host through virulence factors (VFs) [9], which can be acquired through horizontal gene transfer (LGT) facilitated by mobile genetic elements (MGEs) like complex transposons, genomic islands (GIs), or phages [10].

Currently, there is no effective vaccine against *R. equi*, and only antibiotic combinations are used for treatment [11]. The transfer of antimicrobial resistance genes (ARGs) via MGEs among organisms contributes to the emergence of drug-resistant *R. equi* strains, posing challenges in combating the infection [12, 13]. MGEs provide bacterial pathogens with the ability to acquire traits that facilitate adaptation to changing conditions, including vaccinations, antibiotics, new hosts, or new environments, potentially influencing the evolution of pathogenesis [14].

In recent years, advancements in sequencing technology and the reduction in sequencing costs have made whole genome sequencing combined with bioinformatics analysis an essential approach for exploring bacterial genomes [15]. *R. equi* BJ13 was isolated in 2013 from a British thoroughbred racehorse breeding farm in Beijing, China. In this study, we performed high-throughput sequencing to assemble and annotate the genome sequence of *R. equi* BJ13. Additionally, we analyzed the virulence-related genes and antibiotic resistance genes present in the genome, providing valuable genomic resources for studying and developing control strategies against *R. equi* BJ13.

## Methods

### Bacterial strains and growth conditions

The *R. equi* BJ13 strain, isolated from a British Thoroughbred racehorse breeding farm in Beijing, China, was obtained from our laboratory as a conservation strain in 2013. The strain was initially inoculated on 5% sheep blood plates for purification culture, and individual colonies were harvested the following day. These colonies were then incubated in liquid TSB medium at 37 °C and 200 rpm until they reached the logarithmic growth phase.

### DNA extraction and quality assessment

Bacterial cell precipitates were collected, and genomic DNA was extracted through centrifugation using the Tiangen Bacterial DNA Extraction Kit (Tiangen Bio, Beijing, China) according to the manufacturer’s instructions. The quantification of genomic DNA was performed using a Nanodrop 2500 spectrophotometer (Thermo Fisher Scientific, USA), while the integrity of the DNA was assessed via agarose gel electrophoresis. After quality assessment, the samples were sent to Majorbio Bio-Pharm Technology Co. Ltd., Shanghai, China, for genome sequencing.

### Genome sequencing and assembly

The genome sequencing of *R. equi* BJ13 was conducted using the Illumina HiSeq and PacBio sequencing technologies. Each sample provided no less than 100× PacBio sequencing data and 100× Illumina sequencing data. Fastp quality control with PacBio PE library building was applied to the PacBio sequencing data. The assembly of the complete bacterial genome map was accomplished using the Unicycler assembly software [16] for PacBio sequence assembly. During the assembly process, sequence correction was conducted with the aid of pilonjin software to obtain the complete chromosome and plasmid sequences. To ensure a more accurate and complete assembly, the Illumina HiSeq sequencing method was employed to correct DNA base bias.

### Genome prediction

The Glimmer software [17] was utilized to predict the genes on the chromosome, while GeneMarkS [18] was used for plasmid gene prediction. The tRNAs contained in the genome were predicted using tRNAscan-SE v2.0 [19], and the rRNAs were predicted with Barrnap software [20]. Repeatmasker software [21] was employed to identify and classify sequences that showed similarity to known repetitive sequences. BLAST comparisons against a local House-keeping Gene database were conducted to obtain information about the house-keeping genes present in the bacterial genome. Possible sRNA predictions on the bacterial genome were annotated using Infernal software and the Rfam database. Tandem Repeats Finder software [22] was utilized to predict tandem repeat sequences and provide information such as their positions, numbers of repeats, and nucleic acid compositions.

### Genome annotation

The predicted coding genes were functionally annotated against six major databases: NR (Non-Redundant Protein Database), Swiss-Prot, Pfam, EggNOG, GO, and KEGG. NR and Swiss-Prot databases were used to annotate the information of each predicted gene, while Pfam was employed for protein sequence alignment and protein structure domain identification. Gene functions were annotated using the GO database, metabolic pathways were annotated using the Kyoto Encyclopedia of Genes and Genomes (KEGG) database, and predictions of protein function based on homologous sequences were obtained from the EggNOG database.

### Repeat identification and analyses

Various tools were employed for repeat identification and analysis. Island Viewer [23] was used to predict genome islands, Phage_Finder [24] for pre-phage prediction, Minced [25] for CRISPR-Cas prediction, Integron_Finder [26] for integron prediction, ISEScan software [27] for insertion sequence analysis, and TransposonPSI [28] for transposon prediction.

### Analysis of genome circular map

Genome circular maps were created using CGView [29] and Circos [30]. These maps provide a comprehensive overview of gene distribution on the forward and antisense strands, COG functional classification of genes, GC content, genomic islands, and other relevant information.

### Phylogenetic analysis

Phylogenetic analyses were conducted to determine the evolutionary relationships based on 16 S rRNA genes and housekeeping genes. The MEGA 6.0 software [31] was utilized for these analyses, and evolutionary trees were constructed using the Neighbor-Joining (NJ) method.

### Analysis of pathogenic systems

To identify virulence factors, the Virulence Factor Database (VFDB) [32] was used. The prediction of resistance genes was performed using the Comprehensive Antibiotic Resistance Database (CARD) [33]. Pathogen-host interaction genes were predicted using the Pathogen-Host Interactions (PHI) Database [34]. For the analysis of secretory system genes, alignment with the KEGG database was conducted using the Diamond matching software. Prediction of secretory proteins was performed using the signalP database [35]. Transporter-related genes were obtained by aligning with the Transporter Classification Database (TCDB) database [36]. Transmembrane protein-related genes were obtained using the Transmembrane helix hidden Markov model (TMHMM) software [37]. Analysis of two-component regulatory systems was conducted by aligning with the Pfam database using the HMMER3 software [38].

### Experimental animals

Twelve 6-week-old female BALB/c mice (purchased from Spafas (Beijing) Biotechnology Co., Ltd.) were acclimatized to the new environment for 7 days prior to the start of the experiment. The mice were randomly divided into two groups, with six mice in each group. The control group (Con) received an intraperitoneal injection of µL PBS, while the *R. equi* infection group received an intraperitoneal injection of 100 µL of a bacterial suspension containing 5 × 10^7 colony-forming units (CFU)/mL. Throughout the experiment, the mice had ad libitum access to commercial rodent chow and water. A 12-hour light-dark cycle was maintained during the feeding period. All animal experiments were conducted in animal biosafety level 2 (ABSL2) conditions at the Institute of Zoology, Chinese Academy of Sciences. The survival rate and body weight of the mice were monitored for 8 days. At the end of the experiment, the animals were anesthetized with isoflurane, followed by euthanasia after they were fully unconscious to minimize suffering, and blood samples as well as organ tissues (lungs, liver, and spleen) were collected.

### H&E staining of intestinal tissues

After euthanizing the mice, the abdominal cavity was opened, and the overall condition of the intestine was observed. Tissue samples measuring 0.5–1 cm from the colon, below the cecum, were taken, fixed in 10% polyformaldehyde, and stored. Paraffin sections were prepared and stained with Hematoxylin and eosin (H&E) staining for pathological examination of the mouse colon tissue.

### ELISA analysis of cytokine concentrations

To determine the cytokine levels, the blood were subjected to clotting overnight at 4 °C. Subsequently, centrifugation was performed at 1000 rpm for 5 min at 4 °C to separate the serum. The concentrations of cytokines, including TNF-α, IL-1β, IL-4, IL-6, IL-10, and IFN-γ, were measured using the ABplex Mouse Cytokine 6-Plex Assay Kit (ABclonal) (Catalog number: RK04328).

## Results

### Genome sequencing features

The genome sequencing of *R. equi* strain BJ13 was performed using PacBio and Illumina HiSeq sequencing technologies. From the PacBio sequencing, a total of 1,596,402 reads were obtained, with a total length of 13,225,518,821 bp. The longest read obtained was 175,219 bp, and the average read length was 8,284.58 bp. From the Illumina HiSeq sequencing data, a total of 1,548,095,488 bp of raw bases and 1,536,893,592 bp of clean bases were obtained. Additionally, 54,029 clean single reads were obtained from the Illumina sequencing.

**Fig. 1:**
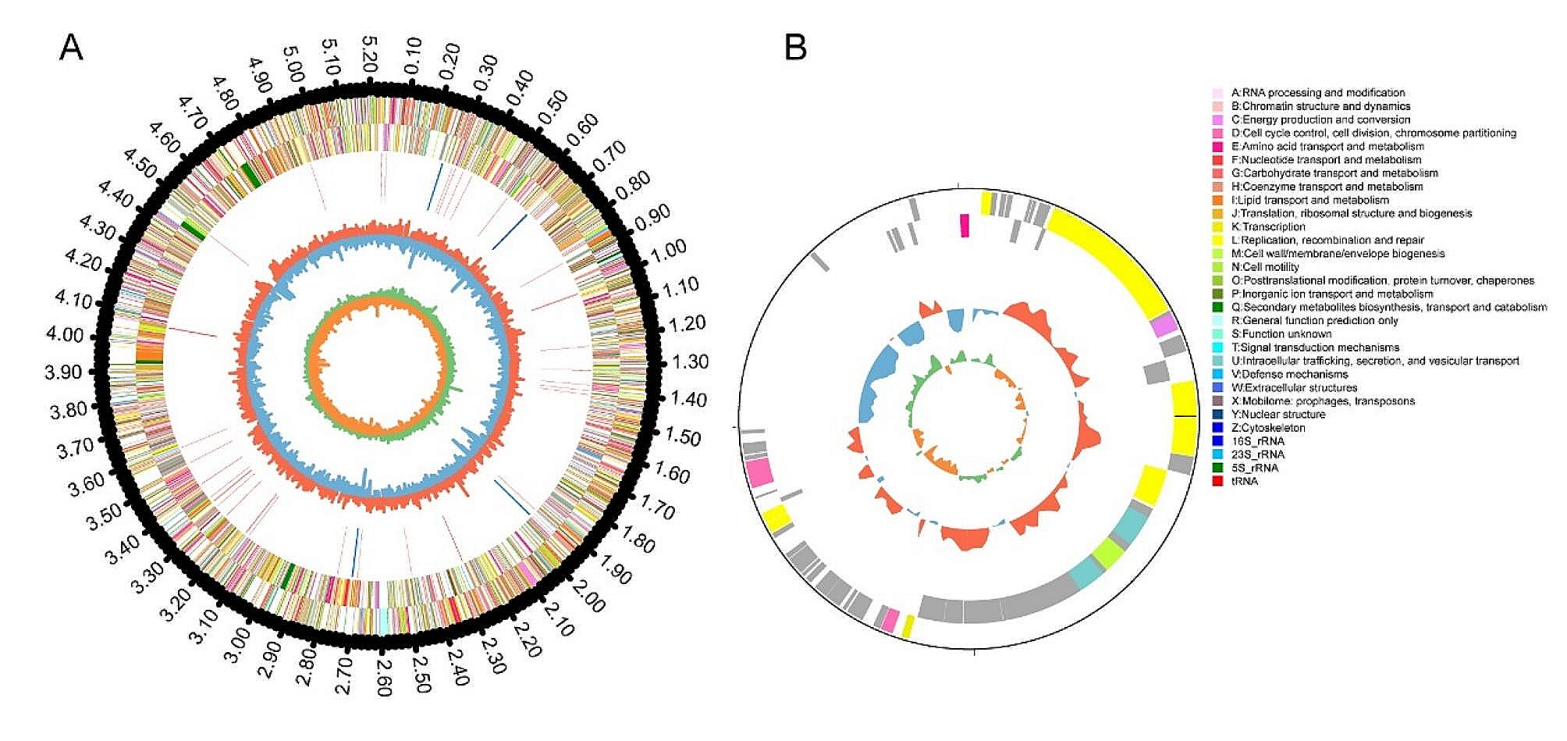
Genome circular map of R equi BJ13 (PRJNA931239). The genome consists of **(A)** a Chromosome and **(B)** a Plasmid. The five layers in order from the outmost to the innermost represent: (1) genome indicated by lengths of DNA sequences in kb, (2) and (3) coding sequences (CDS) on the positive strand and negative strand, respectively, with color bars representing clusters of orthologous group (COG) classifications, (4) locations of rRNA and tRNA genes, (5) GC contents with red bars and blue bars proportionally indicating areas with GC contents higher and lower than the average GC content of the entire genome, respectively, and (6) values of GC-skew as calculated using the algorithm of G − C/G + C

The PacBio reads were used for de novo assembly using the Hierarchical Genome Assembly Process. The assembled genome of *R. equi* strain BJ13 had a size of 5,302,846 bp and a GC content of 68.68%. It consisted of a single circular chromosome of 5,222,239 bp with a GC content of 68.74% and a plasmid of 80,607 bp with a GC content of 64.62% (Fig. 1A, B).

The genome sequence of strain BJ13 has been deposited in the NCBI database under the accession number PRJNA931239. Based on the 16 S rRNA genes (Fig. S1A) and housekeeping genes (Fig. S1B), an evolutionary analysis was conducted with the 20 closest strains at the species level, revealing that *R. equi* strain BJ13 is most closely related to *Prescottella equi* strain DSM 20,295 (NCBI RefSeq assembly: GCF_001646645.1) in terms of phylogenetic evolution.

### Genome prediction and annotation

A total of 4,929 coding gene open reading frames (ORFs) were predicted in the genome of *R. equi* strain BJ13, with a combined length of 4,824,930 bp. These coding genes had an average length of 978.89 bp, accounting for 90.99% of the genome. The GC content in the gene region was calculated to be 69.14% (Table 1).

**Table 1:** Molecular characteristics of the genome of *R. equi* strain BJ13

Length	5,302,846 bp
Total number of coding genes (ORFs)	4929
Total length of coding genes	4,824,930 bp
Average length of coding genes	978.89 bp
Coding genes as a percentage of the genome	0.9099
GC content in the gene region	0.6914
No. of Interpersed Repeat	19
Number of tRNAs predicted	52
Number of rRNAs predicted	12
Number of housekeeping genes	27
Number of sRNAs predicted	26
Total length of sRNAs	3,423 bp (0.0646% of the entire genome)
Total number of repeats predicted	84
Cumulative length of repeats	11,910 bp (0.25% of the entire genome)
Number of interspersed repeats	19 (14 SINEs, 4 LINEs, 1 transposon)
Length of interspersed repeats	1,218 bp (0.03% of the entire genome)

Functional annotation was performed on the predicted 4,929 coding genes in the genome of *R. equi* strain BJ13 by aligning them with 6 databases: NR, Swiss-Prot, Pfam, EggNOG, GO, and KEGG. Genes were functionally annotated using these databases, with NR annotating 4,921 genes, Swiss-Prot annotating 3,569 genes, Pfam annotating 4,103 genes, COG classifying 3,910 genes, GO categorizing 2,921 genes, and KEGG assigning 2,335 genes to biological pathways. Among these databases, GO, KEGG, and COG annotations were particularly informative, covering 59.26%, 47.37%, and 79.33% of the coding genes, respectively.

**Fig. 2:**
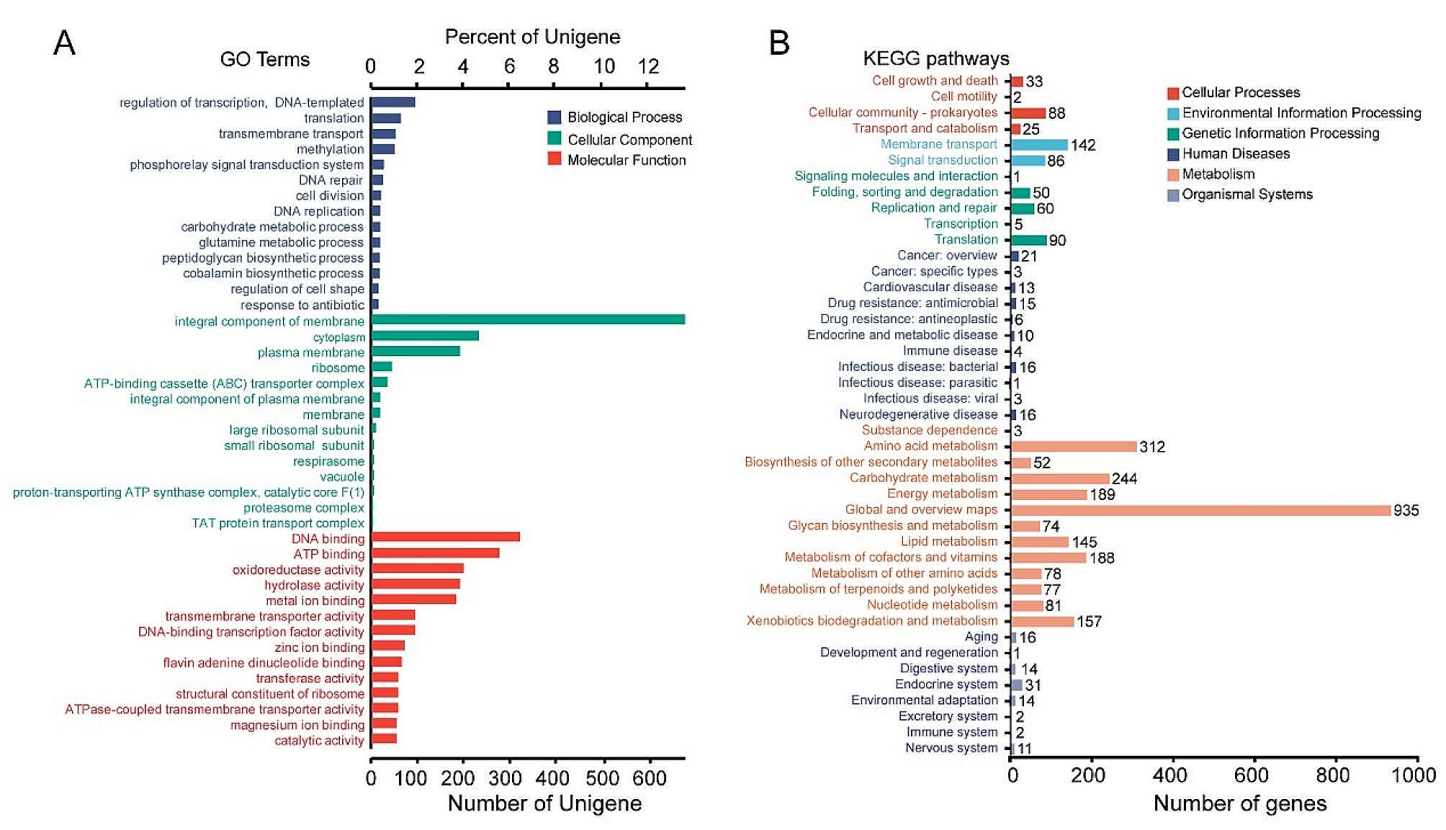
Genome annotation results. **(A)** Content distribution of functional genes from R equi BJ13 based on the Gene Ontology (GO) database; **(B)** Categories of metabolic pathways of genes from R equi BJ13 based on the Kyoto Encyclopedia of Genes and Genomes (KEGG) database

The COG database classified the 3,910 coding genes into 24 categories, with the highest number of annotations in three COG types: general function prediction only (457 genes), transcription (447 genes), and lipid transport and metabolism (440 genes) (Fig. S2). In the GO database, 2,921 genes out of 4,929 ORFs were annotated, with the majority categorized as molecular function (MF) genes (2,431 genes), followed by biological process (1,148 genes) and cellular component (1,089 genes) genes (Fig. 2A).

**Fig. 3:**
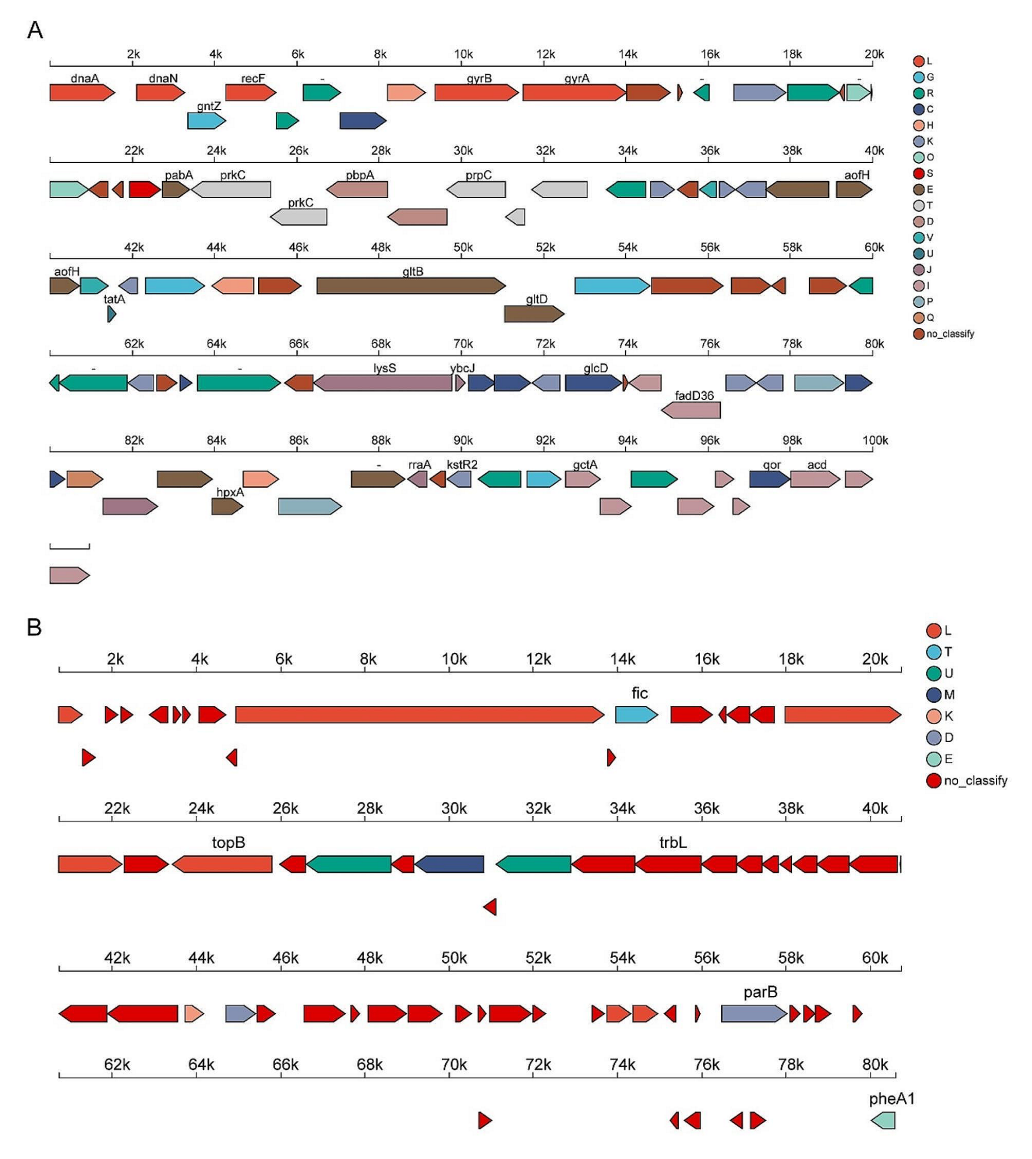
Analysis of Linear Genome Plot. **(A)** Linear Genome Plot of Chromosome. **(B)** Linear Genome Plot of plasmid. The length and direction of the arrows on the map represent the length and coding direction of the gene, respectively, and the color of the arrows represents the COG classification of the gene, with the name of the gene annotated by KEGG below the arrow

Additionally, 2,335 coding genes out of the 4,929 ORFs were annotated across 43 biological pathways in the KEGG database (Fig. 2B). The metabolism pathway, particularly the global and overview maps, had the highest number of annotated genes (935 genes), whereas development and regeneration, infectious disease: parasitic, and signaling molecules and interaction pathways had the lowest number of annotated genes, with only one coding gene assigned to each pathway. Based on the annotation results, we constructed Linear Genome Plot for both the chromosome (Fig. 3A) and the plasmid (Fig. 3B), with gene annotations indicating the coding directions.

### Analysis of mobile genetic elements

A total of 6 genomic islands were predicted in the genome of *R. equi* strain BJ13, with 5 located on the chromosome and one on the plasmid. The coding regions of these genomic islands were aligned with the NR database, resulting in the prediction of 156 functional proteins (Table S1). Using Phage_Finder, a prophage gene located on the chromosome was predicted, potentially derived from Siphoviridae. This prophage gene has a total length of 29,982 bp, containing 49 coding sequences (CDs). Four CRISPR-Cas systems were predicted using Minced in this study. In the plasmid, an insertion sequence spanning 1,538 bp was identified, consisting of two coding regions (Table S2). Additionally, integrons were predicted using the Integron_Finder software, but none were found in the genome.

### Analysis of pathogenic system

**Fig. 4:**
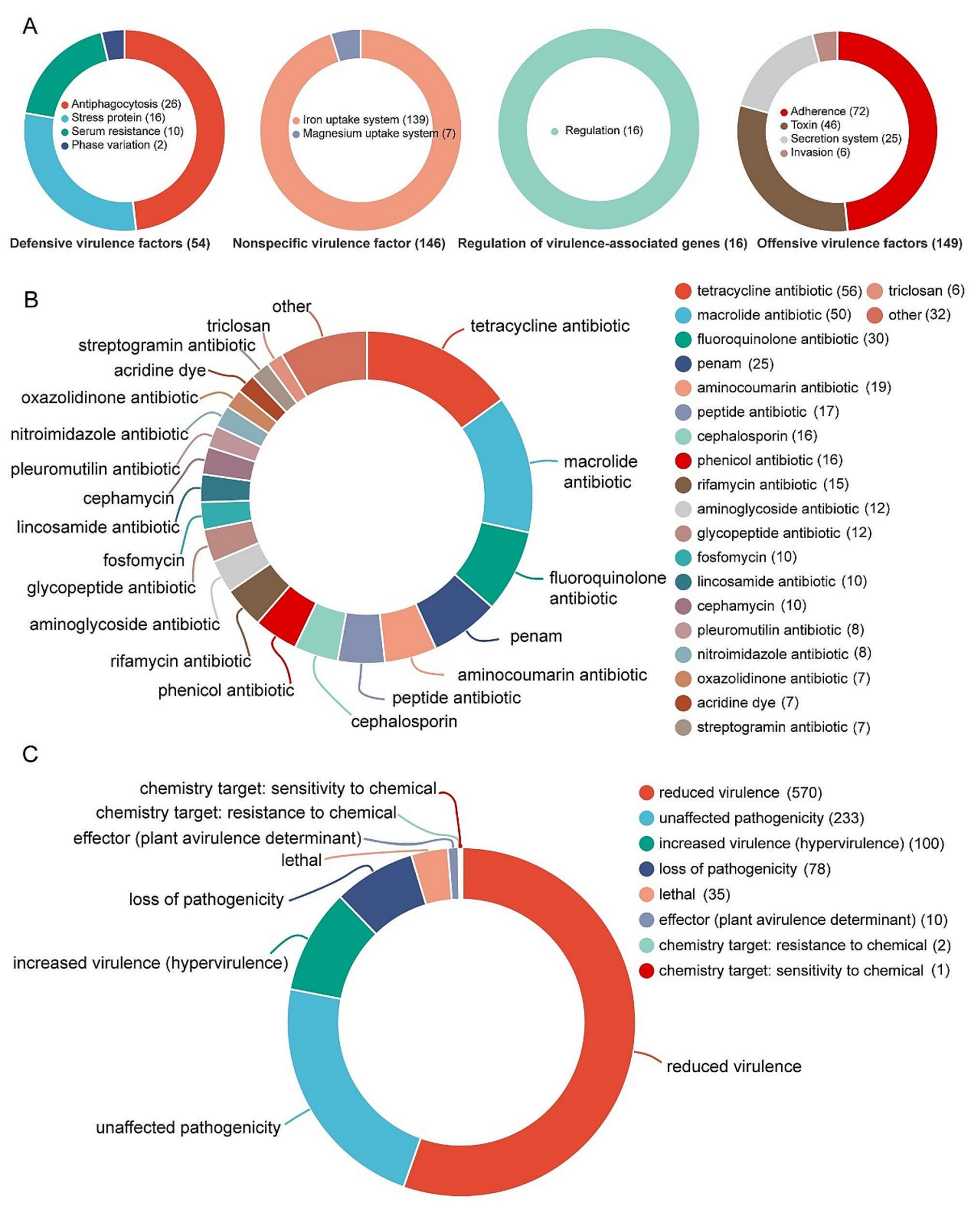
Analysis of pathogenic system. **(A)** Statistical map of predicted classification of virulence genes of BJ13. The text above the circle chart is the primary classification of virulence factors, and the text to the right of the circle chart is the secondary classification. Different colors represent different secondary classifications, and their area indicates the relative proportion of genes in that classification; **(B)** Statistical chart of drug resistance gene prediction classification; **(C)** Statistical map of pathogen host interaction gene classification

Using the VFDB database, a total of 418 virulence factors (VFs) were predicted in the genome of *R. equi* strain BJ13, classified into 4 primary classifications and 11 secondary classifications (Table S3). Among the identified VFs, 54 were categorized as Defensive virulence factors, with Antiphagocytosis genes (26) being the most prevalent. The most abundant group among the genes encoding Nonspecific virulence factors was the Iron uptake system virulence factors, comprising 139 VFs and accounting for 33.25% of the total. Additionally, there were 16 virulence-associated genes and 149 Offensive virulence factors, including 72 Adherence virulence factors (Fig. 4A). The CARD database contains reference genes associated with antibiotic resistance from various organisms, genomes, and plasmids. Through annotation of the CARD database, a total of 225 drug resistance genes were identified across 31 Drug Classes (Table S4). Among the top 20 categories selected for presentation, tetracycline antibiotics had the highest count of 56, followed by macrolide antibiotics with 50 (Fig. 4B). Through annotation of the PHI database, the genes involved in pathogen-host interactions within the BJ13 genome were identified. A total of 900 genes influencing 8 phenotypes were predicted to affect the outcome of pathogen-host interactions (Table S5). The majority of genes were associated with reduced viral phenotypes (570 genes), followed by increased virus (hypervirulence) genes (100 genes), unaffected pathogenicity genes (233 genes), loss of pathogenicity genes (78 genes), and lethal genes (35 genes) (Fig. 4C).

### Analysis of secretory system

15 genes involved in the secretion system were predicted in *R. equi* strain BJ13, with 11 belonging to the Sec-SRP Secretion System and 4 belonging to the Tat Secretion System (Table S6). The signal peptide prediction tool signalP was utilized to predict 297 secreted proteins. Using the TCDB transport protein classification database, a total of 750 transport proteins were predicted. The TMHMM software identified 1,106 transmembrane protein-related genes in the samples. By utilizing the HMMER3 software, we predicted 85 gene pairs associated with the two-component regulatory system. Among these pairs, 54 encoded regulators and 31 encoded sensors, while no genes encoding hybrids were identified.

### Pathogenicity analysis of *R. equi* infection in mice

**Fig. 5:**
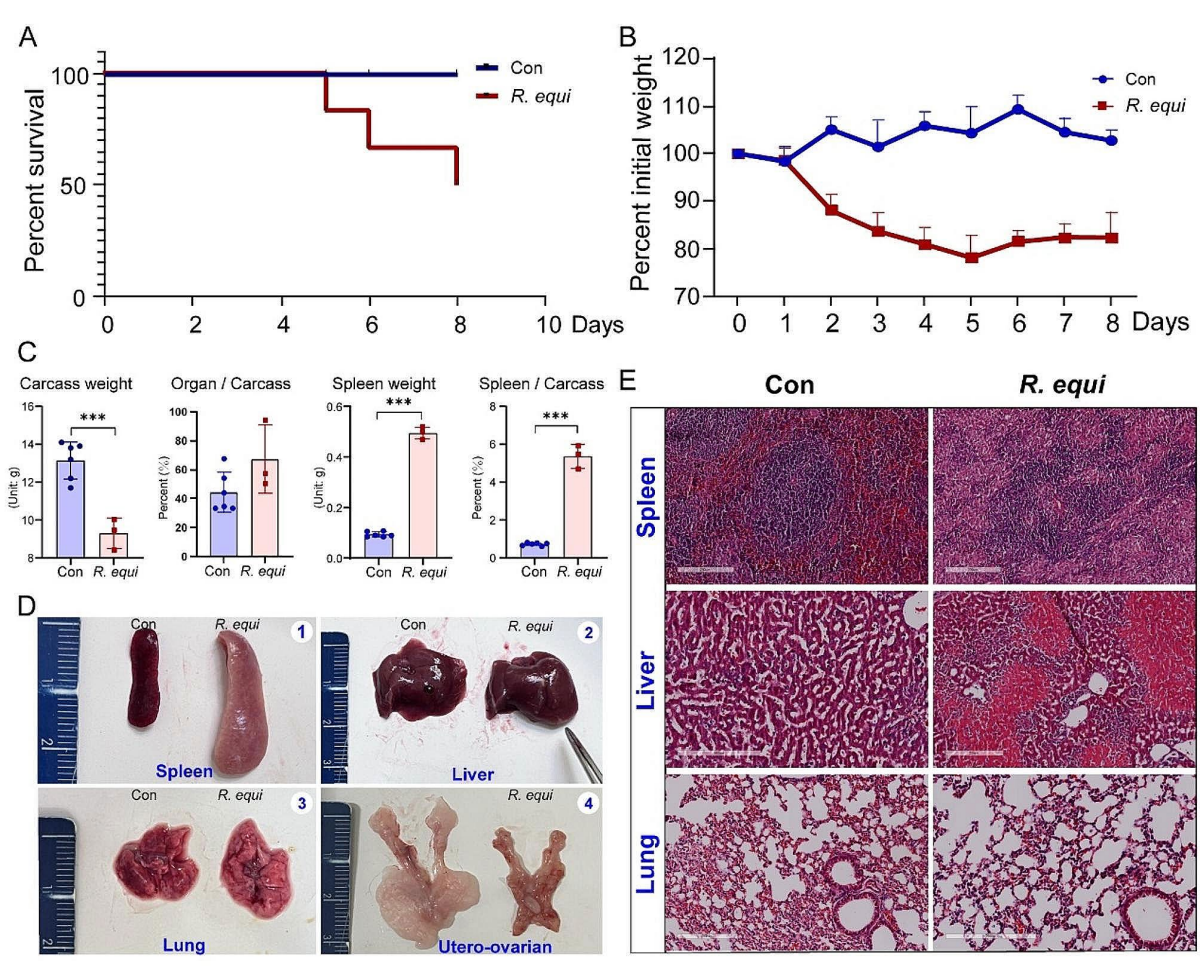
Illustrates the impact of *R. equi* infection on the pathological condition of mice. **(A)** Survival rate of mice; **(B)** Changes in body weight; **(C)** Physiological parameters such as carcass weight and organ weight; **(D)** Observation of changes in the spleen, liver, lungs, and reproductive organs of mice on the 8th day after bacterial infection, Using ordinary one-way analysis of variance (ANOVA) for multiple comparisons. *, **, and *** represent *P* < 0.05, *P* < 0.01, and *P* < 0.001, respectively; (E) Pathological results of lung tissue observation in mice on the 8th day after bacterial infection

During the first 1–2 days after infection, mice in *R. equi* infection group exhibited reduced appetite and water intake, decreased activity levels, increased sensitivity to external stimuli, slight nervousness, and a tendency to aggregate; whereas the Con group maintained normal eating habits and physical condition. On the 5th day, there was mortality observed in *R. equi* infection group mice (16.7%, 1/6), and by the 8th day of the experiment, the mortality rate reached 50% (3/6) (Fig. 5A). Statistical analysis was conducted on the body weight of the experimental mice after infection, and the results revealed a significant decrease in body weight following *R. equi* infection, which ceased to decline by the 5th day (Fig. 5B). There was a significant difference in body weight between the two groups, with an increase in the total organ index and an increase in spleen weight and organ index in the *R. equi* infection group (Fig. 5C).

Upon dissection, it was observed that the spleens of mice in *R. equi* infection group were enlarged, measuring 2.3 cm in length and 0.7 cm in width. The spleen edges appeared blunt and round, with a slight outward folding on the cut surface. The texture was slightly congested, and the color turned pale, without any signs of bleeding or necrotic areas. In contrast, the spleens of mice in Con group did not exhibit any apparent macroscopic abnormalities. The H&E of spleen revealed that the lymphoid follicles and perivascular lymphoid tissue in the spleens of infected mice were more scattered, with a higher number of lymphocytes in the red pulp region, while the white pulp was significantly reduced. The livers of mice in the *R. equi* infection group exhibited blunted edges, enlargement, and visible hemorrhagic spots and necrotic lesions. H&E staining of liver tissue showed significant congestion in the veins and sinusoids of infected mice, with incomplete arrangement of hepatic cords. There was a substantial infiltration of inflammatory cells, varying degrees of granular degeneration, cell shrinkage, and hemorrhagic congestion. Macroscopically, there were no pathological changes observed in the lungs. However, the H&E staining of lung tissue showed evident mild to moderate diffuse infiltration of inflammatory cells, slight epithelial cell degeneration in the alveoli, and mild epithelial cell degeneration in the bronchi. Surprisingly, we also discovered reproductive system damage in the *R. equi* infection group, including impaired development of bilateral ovaries, shortened uterine horn length, and most notably, reduced surrounding adipose tissue with evident hemorrhage (Fig. 5D, E). Thus, it is evident that *R. equi* infection exhibits significant differences in pathogenicity among organs such as the spleen, liver, and lungs.

**Fig. 6:**
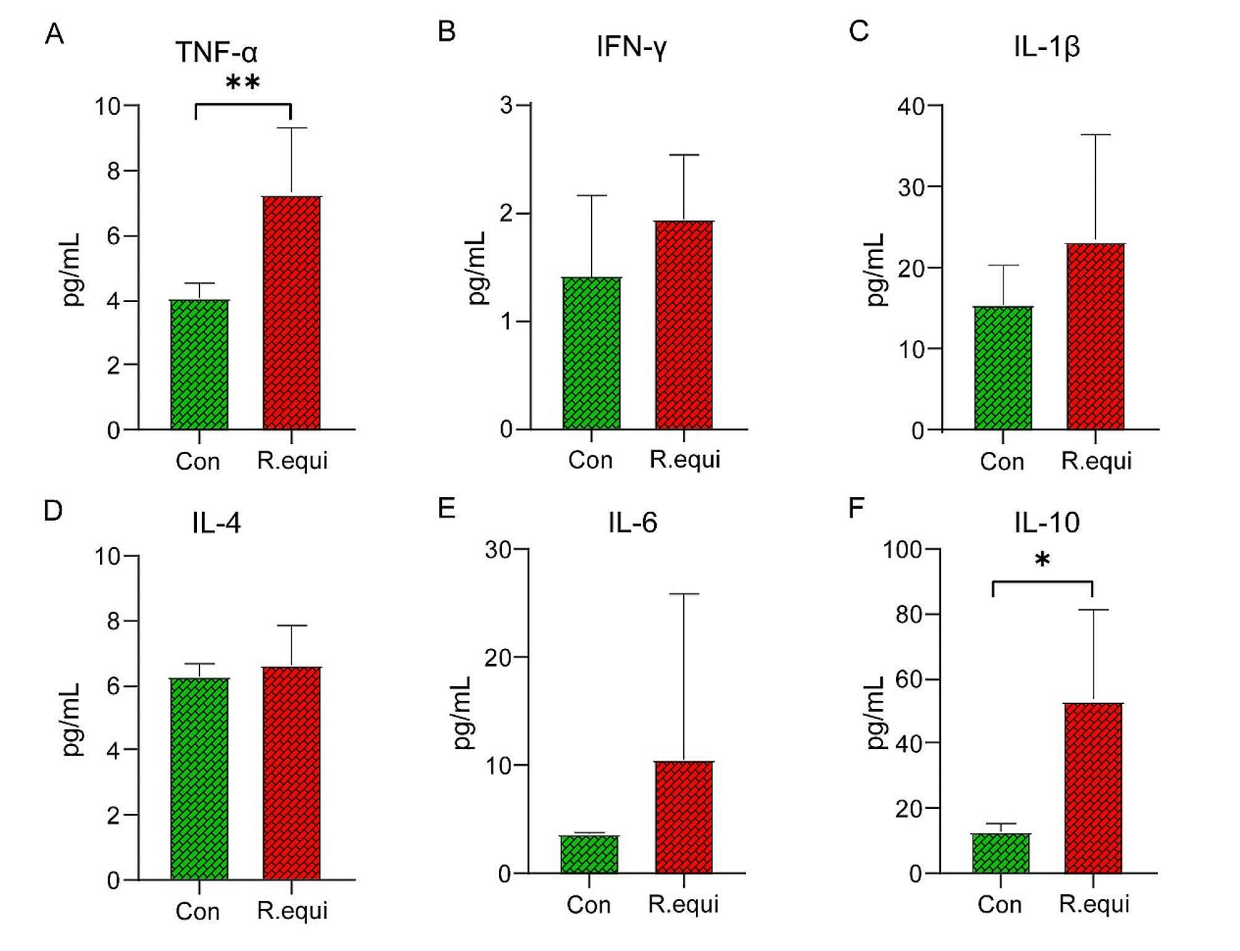
Concentrations of cytokines in mouse intestinal serum. The concentrations of **(A)** TNF-α, **(B)** IFN-γ, **(C)** IL-Iβ, **(D)** IL-4, **(E)** IL-6 and **(F)** IL-10 in the serum were measured. Multiple comparisons were performed using ordinary one-way analysis of variance (ANOVA). *, **, and *** represent *P* < 0.05, *P* < 0.01, and *P* < 0.001, respectively

In order to further analyze the immune response of mice to *R. equi* infection, we measured the concentrations of various cytokines including TNF-α, IFN-γ, IL-Iβ, IL-4, IL-6, and IL-10 in the serum. Compared to the Con group treated with PBS orally, the concentrations of TNF-α (*P*<0.01) and IL-10 (*P*<0.05) in *R. equi* infection group were significantly increased (Fig. 6). Although there was an increase in the concentrations of IFN-γ, IL-Iβ, IL-4, and IL-6, there were no significant statistical differences observed. This suggests that *R. equi* infection may lead to elevated expression of inflammatory factors in mice, thereby triggering a robust immune response.

## Discussion

In this study, we conducted whole-genome sequencing of *R. equi* BJ13 to analyze and characterize its pathogenic and secretory systems. The GC content of BJ13 was determined to be 68.68%, which is consistent with previously reported strains (Ma et al., 2020). BJ13 possesses a total of 4,929 coding genes [39].

Mobile genetic elements (MGEs) play a crucial role in bacterial adaptation to environmental changes and enhancing their competitiveness. Genomic islands (GIs) are important forms of MGEs involved in horizontal transfer. For instance, we identified an Nfed-like membrane protein on the first GI of the chromosome, potentially contributing to maintaining membrane integrity under cell stress conditions [40]. Additionally, the phage integrase on the third GI of the chromosome facilitates gene integration between phage DNA and bacterial DNA, playing a vital role in phage-bacteria interactions [41]. SAM-dependent methyltransferase, present in BJ13, is involved in diverse biological reactions, including DNA methylation [42]. Furthermore, a protein encoding a type II toxin-antitoxin system, specifically the prevent-host-death family antitoxin on the fifth GI, may be associated with the bacterium’s pathogenicity [43].

Prophages in BJ13 might also contribute to its pathogenicity. For example, a protein encoding a WhiB family transcriptional regulator has been implicated in the virulence and antibiotic resistance of *Mycobacterium* and *Corynebacterium* [44]. N-acetylmuramoyl-L-alanine amidase, or autolysin, plays a critical role in peptidoglycan structure dissolution and bacterial cell wall homeostasis [45]. Autolysins are involved in bacterial infection, host intracellular survival, and the regulation of peptidoglycan degradation and wall peptide release [46, 47]. The single-stranded DNA-binding protein is essential for DNA replication, recombination, and repair by specifically binding to single-stranded DNA regions [48].

Among the virulence factors (VFs) identified, Antiphagocytosis genes constitute the largest subgroup of defensive virulence factors, potentially contributing to *R. equi* BJ13’s survival within macrophages [49]. Furthermore, the Iron uptake system virulence factor accounts for a significant proportion (33.25%) of nonspecific VFs. This characteristic is common among many pathogens as it allows efficient iron uptake and enhances competitiveness with the host for this essential element [50].

Our analysis also revealed the presence of macrolide antibiotic resistance genes in the BJ13 genome, consistent with previous laboratory detection tests [51]. In our investigation of pathogen-host interactions, we observed the highest number of genes associated with reduced virulence (570 genes) compared to created virulence (hypervirulence) genes (100 genes). This suggests that BJ13 may exhibit a trend towards reduced virulence, but further experiments are required to confirm this hypothesis.

In this study, we found that the pathogenicity of *R. equi* infection varies among different organs. Previous studies have also indicated that *R. equi* infection can cause lesions in the lungs, liver, and spleen [52]. Our research revealed that the spleen is the organ most visibly affected by the infection, possibly due to its role as the largest immune organ in the body, which may lead to splenomegaly under immunostimulation [53]. Furthermore, different modes of administration may result in varying patterns of organ lesions. *R. equi* can cause pulmonary lesions and even mortality in mice through respiratory infection [54]. In our experiments, the liver and spleen tissues of mice exhibited more severe lesions, possibly due to the selected intraperitoneal infection method, where bacteria can directly enter the liver and spleen of mice via intraperitoneal infection and undergo immune attack. Bacteria entering the bloodstream are also captured by macrophages in the liver and spleen, thus increasing the bacterial load in these organs [55].

Moreover, *R. equi* has the ability to enter and survive within host cells. Once inside host macrophages or other cells, it can evade digestion and killing, utilizing host cells as a favorable environment for its survival and replication. The production of Th1 cytokines is crucial for clearing intracellular pathogens, which is also associated with the clearance of *R. equi* [56]. Among Th1 cytokines, TNF-α is a typical pro-inflammatory cytokine, excessive production of which is associated with host tissue damage [57]. Excessive expression of TNF-α may be attributed to the activated immune response against *R. equi* infection, promoting inflammation and host defense mechanisms.

Consistent with other studies, elevated expression of IL-10 was observed in this study as well [58]. IL-10 is an immunosuppressive cytokine that regulates and limits inflammatory responses during inflammation processes, promoting immune tolerance to prevent excessive immune damage [59]. However, certain pathogens, including *R. equi*, exploit this ability to suppress host immune responses, enabling them to evade immune attacks and survive within the host, thereby promoting their survival and dissemination [60]. IL-10 can inhibit the production of inflammatory cytokines such as tumor necrosis factor-alpha (TNF-α), interferon-gamma (IFN-γ), and interleukin-6 (IL-6) by macrophages and other immune cells. These cytokines play important regulatory roles in immune responses, including activating and enhancing the bactericidal capacity of macrophages, promoting T cell activation and proliferation, etc. [61]. However, due to the short duration of infection and inadequate number of experimental mice, the differences were not significant, indicating the need for further research.

## Conclusion

*R. equi* strain BJ13 exhibits distinct genomic signatures, virulence-associated genes, potential drug resistance, and virulence plasmid structures that may contribute to the evolution of its pathogenicity. *R. equi* employs diverse virulence factors and mechanisms to establish its pathogenicity, which interact and synergize to cause infection and disease. *R. equi* infection elicits intricate immune regulatory mechanisms, involving overexpression of IL-10 and enhanced production of pro-inflammatory cytokines like TNF-α. The delicate balance of these immune responses holds substantial implications for the infection progression, host defense, and may play a pivotal role in disease development and treatment.

### Electronic supplementary material

Below is the link to the electronic supplementary material.

Supplementary Material 1

## Data Availability

All analyzed data are included in the supplementary Excel tables. The data presented in the study are deposited in the National Center for Biotechnology Information (NCBI), accession number PRJNA931239.

## References

[1] Pal M, Rahman T. Rhodococcus equi: an emerging zoonotic pathogen. Ann Vet Anim Sci. 2015;2:3–10.

[2] Vázquez-Boland JA, Meijer WG. The pathogenic actinobacterium Rhodococcus equi: what’s in a name? Mol Microbiol. 2019;112(1):1–15. 31099908 10.1111/mmi.14267 PMC6852188

[3] Giguère S, Cohen ND, Chaffin MK, Hines SA, Hondalus MK, Prescott JF, Slovis NM. Rhodococcus equi: clinical manifestations, virulence, and immunity. J Vet Intern Med. 2011;25(6):1221–30. 22092609 10.1111/j.1939-1676.2011.00804.x

[4] Lin WV, Kruse RL, Yang K, Musher DM. Diagnosis and management of pulmonary infection due to Rhodococcus equi. Clin Microbiol Infect. 2019;25(3):310–5. 29777923 10.1016/j.cmi.2018.04.033

[5] Zink MC, Yager JA, Prescott JF, Fernando MA. Electron microscopic investigation of intracellular events after ingestion of Rhodococcus equi by foal alveolar macrophages. Vet Microbiol. 1987;14(3):295–305. 3672872 10.1016/0378-1135(87)90117-9

[6] Álvarez-Narváez S, Huber L, Giguère S, Hart KA, Berghaus RD, Sanchez S, Cohen ND. Epidemiology and molecular basis of Multidrug Resistance in Rhodococcus equi. Microbiol Mol Biol Rev 2021, 85(2). 10.1128/MMBR.00011-21 PMC8139527 33853933

[7] Ribeiro MG, Lara GHB, da Silva P, Franco MMJ, de Mattos-Guaraldi AL, de Vargas APC, Sakate RI, Pavan FR, Colhado BS, Portilho FVR, et al. Novel bovine-associated pVAPN plasmid type in Rhodococcus equi identified from lymph nodes of slaughtered cattle and lungs of people living with HIV/AIDS. Transbound Emerg Dis. 2018;65(2):321–6. 29226632 10.1111/tbed.12785

[8] Von Bargen K, Haas A. Molecular and infection biology of the horse pathogen Rhodococcus equi. FEMS Microbiol Rev. 2009;33(5):870–91. 19453748 10.1111/j.1574-6976.2009.00181.x

[9] Coulson GB, Agarwal S, Hondalus MK. Characterization of the role of the pathogenicity island and vapG in the virulence of the intracellular actinomycete pathogen Rhodococcus equi. Infect Immun. 2010;78(8):3323–34. 20439471 10.1128/IAI.00081-10 PMC2916281

[10] Valero-Rello A, Hapeshi A, Anastasi E, Alvarez S, Scortti M, Meijer WG, MacArthur I, Vázquez-Boland JA. An Invertron-Like Linear plasmid mediates intracellular survival and virulence in bovine isolates of Rhodococcus equi. Infect Immun. 2015;83(7):2725–37. 25895973 10.1128/IAI.00376-15 PMC4468562

[11] Álvarez-Narváez S, Giguère S, Anastasi E, Hearn J, Scortti M, Vázquez-Boland JA. Clonal confinement of a highly mobile resistance element driven by combination therapy in Rhodococcus equi. mBio 2019, 10(5). 10.1128/mBio.02260-19 PMC6794481 31615959

[12] Giguère S, Berghaus LJ, Willingham-Lane JM. Antimicrobial Resistance in Rhodococcus equi. Microbiol Spectr 2017, 5(5). 10.1128/microbiolspec.arba-0004-2016 PMC11687536 29052538

[13] Kim H, Kim M, Kim S, Lee YM, Shin SC. Characterization of antimicrobial resistance genes and virulence factor genes in an Arctic permafrost region revealed by metagenomics. Environ Pollut. 2022;294:118634. 34875269 10.1016/j.envpol.2021.118634

[14] Song Y, Xu X, Huang Z, Xiao Y, Yu K, Jiang M, Yin S, Zheng M, Meng H, Han Y, et al. Genomic characteristics revealed plasmid-mediated pathogenicity and ubiquitous Rifamycin Resistance of Rhodococcus equi. Front Cell Infect Microbiol. 2022;12:807610. 35252029 10.3389/fcimb.2022.807610 PMC8891757

[15] Zhang C, Hao Q, Zhang Z, Zhang X, Pan H, Zhang J, Zhang H, Sun F. Whole genome sequencing and analysis of Chlorimuron-Ethyl degrading Bacteria klebsiella pneumoniae 2N3. Int J Mol Sci 2019, 20(12). 10.3390/ijms20123053 PMC6627577 31234527

[16] Wick RR, Judd LM, Gorrie CL, Holt KE. Unicycler: resolving bacterial genome assemblies from short and long sequencing reads. PLoS Comput Biol. 2017;13(6):e1005595. 28594827 10.1371/journal.pcbi.1005595 PMC5481147

[17] Delcher AL, Bratke KA, Powers EC, Salzberg SL. Identifying bacterial genes and endosymbiont DNA with glimmer. Bioinformatics. 2007;23(6):673–9. 17237039 10.1093/bioinformatics/btm009 PMC2387122

[18] Besemer J. M Borodovsky 2005 GeneMark: web software for gene finding in prokaryotes, eukaryotes and viruses. Nucleic Acids Res 33 Web Server issue W451–454. 15980510 10.1093/nar/gki487 PMC1160247

[19] Chan PP, Lowe TM. tRNAscan-SE: searching for tRNA genes in genomic sequences. Methods Mol Biol. 2019;1962:1–14. 31020551 10.1007/978-1-4939-9173-0_1 PMC6768409

[20] Lau KJX, Junqueira ACM, Uchida A, Purbojati RW, Houghton JNI, Chénard C, Wong A, Kolundžija S, Clare ME, Kushwaha KK et al. Complete genome sequence of Agrococcus sp. Strain SGAir0287, isolated from Tropical Air Collected in Singapore. Microbiol Resour Announc 2019, 8(32). 10.1128/MRA.00616-19 PMC6687924 31395637

[21] Tarailo-Graovac M. N Chen 2009 Using RepeatMasker to identify repetitive elements in genomic sequences. Curr Protoc Bioinf Chap. 4 41011–141014. 10.1002/0471250953.bi0410s25 19274634

[22] Benson G. Tandem repeats finder: a program to analyze DNA sequences. Nucleic Acids Res. 1999;27(2):573–80. 9862982 10.1093/nar/27.2.573 PMC148217

[23] Bertelli C, Laird MR, Williams KP, Lau BY, Hoad G, Winsor GL, Brinkman FSL. IslandViewer 4: expanded prediction of genomic islands for larger-scale datasets. Nucleic Acids Res. 2017;45(W1):W30–5. 28472413 10.1093/nar/gkx343 PMC5570257

[24] Fouts DE. Phage_Finder: automated identification and classification of prophage regions in complete bacterial genome sequences. Nucleic Acids Res. 2006;34(20):5839–51. 17062630 10.1093/nar/gkl732 PMC1635311

[25] Bland C, Ramsey TL, Sabree F, Lowe M, Brown K, Kyrpides NC, Hugenholtz P. CRISPR Recognition Tool (CRT): a tool for automatic detection of clustered regularly interspaced palindromic repeats. BMC Bioinformatics. 2007;8(1):209. 17577412 10.1186/1471-2105-8-209 PMC1924867

[26] Moura A, Soares M, Pereira C, Leitão N, Henriques I, Correia A. INTEGRALL: a database and search engine for integrons, integrases and gene cassettes. Bioinformatics. 2009;25(8):1096–8. 19228805 10.1093/bioinformatics/btp105

[27] Siguier P, Gourbeyre E, Chandler M. Bacterial insertion sequences: their genomic impact and diversity. FEMS Microbiol Rev. 2014;38(5):865–91. 24499397 10.1111/1574-6976.12067 PMC7190074

[28] Haas B. TransposonPSI: an application of PSI-Blast to mine (retro-) transposon ORF homologies. *Broad Institute, Cambridge, MA, USA* 2007.

[29] Stothard P, Wishart DS. Circular genome visualization and exploration using CGView. Bioinformatics. 2005;21(4):537–9. 15479716 10.1093/bioinformatics/bti054

[30] Krzywinski M, Schein J, Birol I, Connors J, Gascoyne R, Horsman D, Jones SJ, Marra MA. Circos: an information aesthetic for comparative genomics. Genome Res. 2009;19(9):1639–45. 19541911 10.1101/gr.092759.109 PMC2752132

[31] Kumar S, Stecher G, Tamura K. MEGA7: Molecular Evolutionary Genetics Analysis Version 7.0 for bigger datasets. Mol Biol Evol. 2016;33(7):1870–4. 27004904 10.1093/molbev/msw054 PMC8210823

[32] Chen L, Zheng D, Liu B, Yang J, Jin Q. VFDB 2016: hierarchical and refined dataset for big data analysis–10 years on. Nucleic Acids Res. 2016;44(D1):D694–697. 26578559 10.1093/nar/gkv1239 PMC4702877

[33] Jia B, Raphenya AR, Alcock B, Waglechner N, Guo P, Tsang KK, Lago BA, Dave BM, Pereira S, Sharma AN, et al. CARD 2017: expansion and model-centric curation of the comprehensive antibiotic resistance database. Nucleic Acids Res. 2017;45(D1):D566–73. 27789705 10.1093/nar/gkw1004 PMC5210516

[34] Urban M, Cuzick A, Rutherford K, Irvine A, Pedro H, Pant R, Sadanadan V, Khamari L, Billal S, Mohanty S, et al. PHI-base: a new interface and further additions for the multi-species pathogen-host interactions database. Nucleic Acids Res. 2017;45(D1):D604–10. 27915230 10.1093/nar/gkw1089 PMC5210566

[35] Petersen TN, Brunak S, von Heijne G, Nielsen H. SignalP 4.0: discriminating signal peptides from transmembrane regions. Nat Methods. 2011;8(10):785–6. 21959131 10.1038/nmeth.1701

[36] Saier MH Jr., Tran CV, Barabote RD. TCDB: the transporter classification database for membrane transport protein analyses and information. Nucleic Acids Res. 2006;34(Database issue):D181–186. 16381841 10.1093/nar/gkj001 PMC1334385

[37] Krogh A, Larsson B, von Heijne G, Sonnhammer EL. Predicting transmembrane protein topology with a hidden Markov model: application to complete genomes. J Mol Biol. 2001;305(3):567–80. 11152613 10.1006/jmbi.2000.4315

[38] Eddy SR. A new generation of homology search tools based on probabilistic inference. Genome Informatics 2009. edn.: Published by Imperial College Press and distributed by World Scientific Publishing CO.; 2009. pp. 205–11. 20180275

[39] Ma Q, Gao X, Bi X, Tu L, Xia M, Shen Y, Wang M. Isolation, characterisation, and genome sequencing of Rhodococcus equi: a novel strain producing chitin deacetylase. Sci Rep. 2020;10(1):4329. 32152368 10.1038/s41598-020-61349-9 PMC7062688

[40] Walker CA, Hinderhofer M, Witte DJ, Boos W, Möller HM. Solution structure of the soluble domain of the NfeD protein YuaF from Bacillus subtilis. J Biomol NMR. 2008;42(1):69–76. 18696230 10.1007/s10858-008-9261-3

[41] Groth AC, Calos MP. Phage integrases: Biology and Applications. J Mol Biol. 2004;335(3):667–78. 14687564 10.1016/j.jmb.2003.09.082

[42] Majumdar S, Gupta U, Chinnasamy HV, Laxmipathy S, Matheshwaran S. Zn(2+)-Induced Conformational Change affects the SAM binding in a mycobacterial SAM-Dependent methyltransferase. ACS Omega. 2022;7(40):35901–10. 36249403 10.1021/acsomega.2c04555 PMC9558604

[43] Kamruzzaman M, Iredell J. A ParDE-family toxin antitoxin system in major resistance plasmids of Enterobacteriaceae confers antibiotic and heat tolerance. Sci Rep. 2019;9(1):9872. 31285520 10.1038/s41598-019-46318-1 PMC6614396

[44] Bush MJ. The actinobacterial WhiB-like (Wbl) family of transcription factors. Mol Microbiol. 2018;110(5):663–76. 30179278 10.1111/mmi.14117 PMC6282962

[45] Rice KC, Bayles KW. Molecular control of bacterial death and lysis. Microbiol Mol Biol Rev. 2008;72(1):85–109. table of contents. 18322035 10.1128/MMBR.00030-07 PMC2268280

[46] Vermassen A, Leroy S, Talon R, Provot C, Popowska M, Desvaux M. Cell wall hydrolases in Bacteria: insight on the diversity of Cell Wall Amidases, glycosidases and peptidases toward Peptidoglycan. Front Microbiol. 2019;10:331. 30873139 10.3389/fmicb.2019.00331 PMC6403190

[47] Humann J, Lenz LL. Bacterial peptidoglycan degrading enzymes and their impact on host muropeptide detection. J Innate Immun. 2009;1(2):88–97. 19319201 10.1159/000181181 PMC2659621

[48] Nowak M, Olszewski M, Śpibida M, Kur J. Characterization of single-stranded DNA-binding proteins from the psychrophilic bacteria Desulfotalea Psychrophila, Flavobacterium psychrophilum, Psychrobacter arcticus, Psychrobacter cryohalolentis, Psychromonas ingrahamii, Psychroflexus torquis, and Photobacterium profundum. BMC Microbiol. 2014;14(1):91. 24725436 10.1186/1471-2180-14-91 PMC3991886

[49] Mourenza Á, Collado C, Bravo-Santano N, Gil JA, Mateos LM, Letek M. The extracellular thioredoxin Etrx3 is required for macrophage infection in Rhodococcus equi. Vet Res. 2020;51(1):38. 32156317 10.1186/s13567-020-00763-3 PMC7063783

[50] Caza M, Kronstad JW. Shared and distinct mechanisms of iron acquisition by bacterial and fungal pathogens of humans. Front Cell Infect Microbiol. 2013;3:80. 24312900 10.3389/fcimb.2013.00080 PMC3832793

[51] Liu H, Wang Y, Yan J, Wang C, He H. Appearance of multidrug-resistant virulent Rhodococcus equi clinical isolates obtained in China. J Clin Microbiol. 2014;52(2):703. 24478520 10.1128/JCM.02925-13 PMC3911302

[52] Weinstock DM, Brown AE. Rhodococcus equi: an Emerging Pathogen. Clin Infect Dis. 2002;34(10):1379–85. 11981734 10.1086/340259

[53] Lewis SM, Williams A, Eisenbarth SC. Structure and function of the immune system in the spleen. Sci Immunol. 2019;4(33):eaau6085. 30824527 10.1126/sciimmunol.aau6085 PMC6495537

[54] Kuskie KR, Smith JL, Wang N, Carter CN, Chaffin MK, Slovis NM, Stepusin RS, Cattoi AE, Takai S, Cohen ND. Effects of location for collection of air samples on a farm and time of day of sample collection on airborne concentrations of virulent Rhodococcus equi at two horse breeding farms. Am J Vet Res. 2011;72(1):73–9. 21194338 10.2460/ajvr.72.1.73 PMC3381359

[55] Jain S, Bloom BR, Hondalus MK. Deletion of vapA encoding Virulence Associated protein A attenuates the intracellular actinomycete Rhodococcus equi. Mol Microbiol. 2003;50(1):115–28. 14507368 10.1046/j.1365-2958.2003.03689.x

[56] Hooper-McGrevy KE, Wilkie Bruce N, Prescott John F. Immunoglobulin G Subisotype Responses of Pneumonic and Healthy, exposed foals and adult horses to Rhodococcus equi Virulence-Associated proteins. Clin Vaccine Immunol. 2003;10(3):345–51. 10.1128/CDLI.10.3.345-351.2003 PMC154967 12738629

[57] Jang D-i, Lee AH, Shin H-Y, Song H-R, Park J-H, Kang T-B, Lee S-R, Yang S-H. The role of Tumor necrosis factor alpha (TNF-α) in Autoimmune Disease and current TNF-α inhibitors in therapeutics. In: Int J Mol Sci vol. 22; 2021. 10.3390/ijms22052719 PMC7962638 33800290

[58] Giguère S, Wilkie Bruce N, Prescott John F. Modulation of Cytokine Response of Pneumonic foals by Virulent Rhodococcus equi. Infect Immun. 1999;67(10):5041–7. 10496876 10.1128/IAI.67.10.5041-5047.1999 PMC96851

[59] Iyer SS, Cheng G. Role of interleukin 10 transcriptional regulation in inflammation and autoimmune disease. Crit Rev Immunol. 2012;32(1):23–63. 22428854 10.1615/CritRevImmunol.v32.i1.30 PMC3410706

[60] Berghaus LJ, Giguère S, Bordin AI, Cohen ND. Effects of priming with cytokines on intracellular survival and replication of Rhodococcus equi in equine macrophages. Cytokine. 2018;102:7–11. 29245049 10.1016/j.cyto.2017.12.011

[61] Kessler B, Rinchai D, Kewcharoenwong C, Nithichanon A, Biggart R, Hawrylowicz CM, Bancroft GJ, Lertmemongkolchai G. Interleukin 10 inhibits pro-inflammatory cytokine responses and killing of Burkholderia pseudomallei. Sci Rep. 2017;7(1):42791. 28216665 10.1038/srep42791 PMC5316963

